# Differences in Fine Root Traits of *Quercus rehderiana* Between Rocky and Nonrock Desertification Forests in Southwest China

**DOI:** 10.1002/ece3.72400

**Published:** 2025-11-18

**Authors:** Xiaolong Bai, Shun Zou, Wanchang Zhang, Tu Feng, Dongpeng Lv, Bin He, Wangjun Li

**Affiliations:** ^1^ College of Ecological Engineering Guizhou University of Engineering Science Bijie Guizhou China; ^2^ Guizhou Province Key Laboratory of Ecological Protection and Restoration of Typical Plateau Wetlands Guizhou University of Engineering Science Bijie Guizhou China; ^3^ Key Laboratory of Digital Earth Science, Aerospace Information Research Institute Chinese Academy of Sciences Beijing China; ^4^ International Research Center of big Data for Sustainable Development Goals Beijing China; ^5^ Key Laboratory of Ecological Microbial Remediation Technology of Yunnan Higher Education Institutes Dali University Dali Yunnan China

**Keywords:** fine roots, morphological traits, nutrients, *Quercus rehderiana*, resource strategies, rock desertification

## Abstract

Fine roots (diameter < 2 mm) play a crucial role in plants' acquisition of soil water and nutrients. Their morphological and chemical traits, which reflect resource utilization strategies, have garnered significant global research attention. However, the extent to which these traits are influenced by environmental conditions remains unclear. In this study, we measured 14 morphological and chemical traits from nine *Quercus rehderiana* individuals in both rocky and nonrocky desertification forest habitats. Our objectives were to identify the key factors shaping fine root traits and elucidate their adaptive strategies in the arid, nutrient‐poor conditions of rocky desertification forest ecosystems. The results revealed that *Quercus rehderiana* fine roots in rocky desertification forests exhibited higher root length (RL), root volume (RV), nitrogen (N), calcium (Ca), and magnesium (Mg) concentrations, along with an elevated nitrogen‐to‐phosphorus ratio (N:P ratio), compared to those in nonrocky desertification forests. Conversely, fine roots in nonrocky desertification forests showed greater phosphorus (P) and potassium (K) concentrations. Correlation analysis indicated that in rocky desertification forests, specific root length (SRL) and specific root area (SRA) were positively correlated with root P concentration, while root N concentration exhibited synergistic relationships with P and K. In contrast, nonrocky desertification forests displayed trade‐offs: SRL and SRA were negatively correlated with RV, root diameter (RD), and root tissue density (RTD), and root carbon (C) concentration was inversely related to Ca and Mg. Principal component analysis further demonstrated that rocky desertification forests favored a resource‐acquisition strategy, characterized by enhanced morphological traits and nutrient concentrations, whereas nonrocky desertification forests leaned toward a resource‐conserving strategy, prioritizing P and K accumulation. These findings highlight that *Quercus rehderiana* adapts to divergent environments through distinct resource allocation mechanisms, with its unique traits in rocky desertification forests underscoring its suitability for ecological restoration efforts.

## Graphical abstract

Fine roots (< 2 mm in diameter) play an important role in plants acquiring soil water and nutrient resources. Their morphological and chemical traits linked to resource strategies have received much attention in the world. However, whether the traits related to resource strategies are affected by environmental conditions or phylogeny is still unclear. In this study, 14 fine root traits (morphological and chemical traits) of *Quercus rehderiana* were measured in 9 individuals in forests with rocky and nonrocky desertification. The objective was to analyze the factors influencing fine root morphological and chemical traits and their strategies of adaptation to arid and poor environments in forests with rocky desertification. The results showed that *Quercus rehderiana* fine roots in forests with rocky desertification had higher root length, root volume, nitrogen concentration, calcium concentration, magnesium concentration, and nitrogen phosphorus ratio than in forests with nonrocky desertification. *Quercus rehderiana* fine roots in forests with nonrocky desertification had a greater phosphorus concentration and potassium concentration than in forests with rocky desertification. The results of principal component analysis showed that forests with rocky desertification tended to show resource‐acquisition strategies with higher fine root morphological traits and nutrient concentrations, while forests with nonrocky desertification tended to show resource‐conserving strategies with greater phosphorus and potassium concentrations. We inferred that the fine root morphological traits are determined by phylogeny, while nutrient concentration is influenced by the availability of soil mineral nutrients.

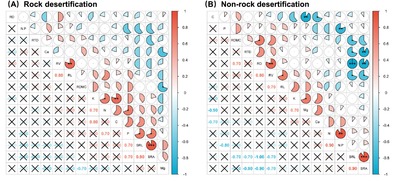

## 1 Introduction

Plant functional traits encompass morphological, physiological, and phenological features that signify plants' adaptive strategies to environmental conditions (Violle et al. 2007). These traits are broadly categorized into leaf, stem, and root system characteristics (Perez‐Harguindeguy et al. 2016). While extensive research has been conducted on leaf functional traits and their environmental responses (Maynard et al. 2022; Niklas et al. 2023), root system traits remain understudied due to methodological challenges in sampling and measurement. This disparity limits comprehensive assessment of plant–environment interactions (Iversen et al. 2017; Kühn et al. 2021).

In terrestrial forest ecosystems, fine roots—typically characterized by a diameter under 2 mm (Artacho and Bonomelli 2016; Valverde‐Barrantes et al. 2015)—perform essential functions in water and nutrient uptake (Comas 2017; Ma et al. 2018; Sun et al. 2021). These specialized root structures demonstrate particular sensitivity to soil conditions, often serving as the primary plant organs responsive to environmental changes (Hendricks et al. 1993). The examination of root functional traits offers valuable understanding of vegetation adaptation mechanisms to soil heterogeneity and patterns of resource investment (Bergmann et al. 2020; Carmona et al. 2021; Kong et al. 2019).

Root functional traits can be systematically classified into four fundamental categories: morphological, chemical, anatomical, and physiological characteristics (Comas et al. 2014; Cusack et al. 2021; Perez‐Harguindeguy et al. 2016). The morphological traits include root length, root diameter, root volume, specific root length, specific root area, root dry matter content, and root tissue density. Chemically, these traits encompass elemental concentrations and stoichiometric ratios (C, N, P, K, Ca, Mg, N:P ratio). Among morphological parameters, root diameter, root volume, and particularly specific root length serve as crucial indicators of resource acquisition potential (Eissenstat 1991; Guo et al. 2008; Mommer and Weemstra 2012; Weigelt et al. 2021). Specific root length quantifies absorption efficiency per unit biomass, with elevated values suggesting more economical resource acquisition (Eissenstat and Duncan 1992). Similarly, specific root area reflects biomass allocation efficiency to absorptive surfaces, where higher values indicate superior nutrient uptake per investment unit (Lõhmus et al. 1989). Root tissue density, representing root mass per unit volume, correlates with growth potential, resource acquisition, and defense strategies (Craine et al. 2001; Eissenstat and Caldwell 1988). Root dry matter content increases in nutrient‐deficient soils as plants prioritize belowground biomass allocation (Díaz et al. 2016). The chemical composition profoundly affects root metabolism, respiration, and turnover rates (Guo et al. 2008; Makita et al. 2009; Ruffel et al. 2011). While C constitutes the fundamental structural component and mediates photosynthetic/respiratory processes (Guo et al. 2008), N and P participate in protein synthesis, nucleic acid production, and metabolic regulation (Lambers, Iii, and Pons 2008; Ruffel et al. 2011). Potassium facilitates osmotic regulation under abiotic stresses (Sardans and Peñuelas 2021; Tränker et al. 2018), whereas Ca and Mg enhance photosynthetic performance and stress tolerance (Verbruggen and Hermans 2013; White and Broadley 2003). Fine root N:P ratios reflect both nutrient cycling capacity and the equilibrium between nutrient availability and plant requirements (Ågren et al. 2012; Sardans et al. 2012; Wang et al. 2019). This integrated trait framework enables plants to respond to environmental gradients (Mommer and Weemstra 2012), underscoring the importance of multivariate trait analysis in deciphering plant‐environment interactions. Particularly under the intense selective pressure imposed by resource limitations in karst rocky desertification ecosystems, understanding the fundamental functional traits of these fine roots is especially critical for plants thriving in such extreme environments, providing a vital scientific basis for the restoration and management of karst rocky desertification.

Karst rock desertification represents a unique geomorphological phenomenon formed through the dissolution of soluble carbonate rocks, with significant occurrences observed in the southern United States, Mediterranean Europe, and southwestern China (Goldscheider et al. 2020; Jiang et al. 2016). Notably, the karst terrain of southwestern China—especially Guizhou Province—comprises one of the most extensive continuous karst landscapes globally (Cao et al. 2004; Wang et al. 2004). These ecosystems exhibit distinctive environmental conditions marked by extensive bedrock exposure, calcareous soils' alkaline properties, elevated temperatures, limited water resources, and shallow soil profiles (Dai et al. 2017; Liu et al. 2022; Wang 2003). The vast expanse of this ecologically fragile region (approximately 550,000 km^2^ in area) exerts profound impacts on both local/regional ecological integrity and socioeconomic development (Li et al. 2022). Furthermore, ongoing climate change and anthropogenic activities continue to exacerbate rocky desertification in certain areas (Li et al. 2022). Statistical data reveal alarming expansion rates exceeding 110 km^2^ a^−1^ in parts of Guizhou Province across different periods (116.2 km^2^ a^−1^ during 1993–2001; 118.58 km^2^ a^−1^ during 2000–2015, Huang and Cai 2007; Li et al. 2022). Consequently, vegetation management and ecological restoration in rocky desertification ecosystems have emerged as critical global environmental challenges, demanding enhanced theoretical research to inform effective mitigation strategies (D'Ettorre et al. 2024). *Quercus rehderiana*, an oak species belonging to the Fagaceae family, demonstrates remarkable ecological adaptability throughout Guizhou Province, where it successfully colonizes nutrient‐deficient soils and water‐limited habitats (Wang et al. 2023). This species exhibits exceptional ecological dominance, serving as the exclusive dominant species (exceeding 80% canopy coverage with uniform distribution) in both rocky and nonrocky desertification forest ecosystems (Bai et al. 2024). Its ability to thrive in both rocky and nonrocky desertification habitats offers a rare opportunity to compare intraspecific adaptive strategies under contrasting soil and microclimatic conditions. This duality allows us to disentangle plasticity from local adaptation, addressing a key knowledge gap in karst rocky desertification ecosystem resilience. Nevertheless, it remains unclear whether this species has developed adaptive traits in rocky habitats (rocky vs. nonrocky) or whether it can meet the requirements for future ecological restoration in rocky regions. To address these questions, our study employs a comparative approach to examine fine root functional traits of *Quercus rehderiana* across rocky desertification and nonrocky desertification habitats. The results will provide valuable scientific guidance for species selection in ecological restoration projects targeting degraded karst landscapes.

## 2 Materials and Methods

### 2.1 Study Site

The research was carried out in Weining County (103°36′–104°30′ E, 26°30′–27°25′ N), situated in Bijie City of northwestern Guizhou Province, China. This region represents a typical mid‐subtropical monsoon climate, with a mean annual temperature of 12°C and precipitation averaging 1000 mm. The study area lies at an average elevation of 2200 m, exhibiting characteristic plateau mountain topography with yellow‐brown soils (pH = 5.50). Anthropogenic disturbances and grazing pressure have resulted in relatively sparse vegetation, featuring a shrub layer dominated by *Rhododendron simsii*, *Cotoneaster franchetii* , and *Corylus yunnanensis*, along with an herbaceous layer comprising *Rubia cordifolia*, *Viola philippica*, and *Plantago asiatica* .

### 2.2 Experimental Design and Sampling

**FIGURE 1:**
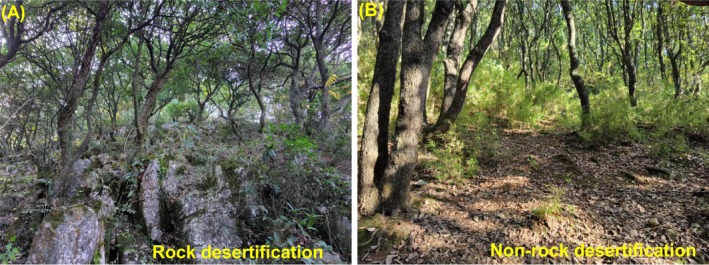
Rock desertification and nonrock desertification forests in the study area.

Rocky and nonrocky desertification areas were distinguished based on vegetation coverage, rock exposure rate, and mean soil thickness (Li et al. 2004). Rocky desertification was defined by three key criteria: vegetation coverage below 35%, rock exposure exceeding 60%, and soil thickness under 15 cm. In contrast, nonrocky desertification forests exhibited vegetation coverage above 35%, rock exposure below 60%, and soil thickness greater than 15 cm. From July to September 2021, five 20 × 20 m quadrats were established in both rocky and nonrocky desertified forests (Figure 1). Within these quadrats, two *Quercus rehderiana* individuals (20–30 cm diameter at breast height) were selected for fine root sampling. However, in one rocky desertification quadrat, only a single *Quercus rehderiana* could be sampled due to the complete encasement of tree roots in bedrock. Consequently, a total of nine individuals were collected across all quadrats. Fine roots (≤ 2 mm diameter) were carefully excavated using the radial trenching method: surface soil (0–30 cm depth) was systematically removed along the four cardinal directions (East, West, South, North) from the tree base. Sieved roots were immediately placed in labeled zip‐lock bags, stored under refrigeration, and transported to the laboratory for subsequent analysis.

### 2.3 Trait Measurements

The fine root traits analyzed in this study are summarized in Table 1. Prior to analysis, fine root samples were rinsed with tap water to remove the attached soils and residues, followed by three distilled water washes. Surface moisture was blotted using absorbent paper before measuring root weight with a precision electronic balance (0.0001 g). Subsequently, roots were immersed in distilled water and scanned at 1200 dpi resolution using a Microtek ScanMaker i850 (Gao et al. 2021). After scanning, samples were oven‐dried at 65°C for 72 h and reweighed. Morphological traits—including RL (cm), RD (mm), RV (cm^3^), and root surface area (cm^2^) − were quantified using DJ‐GX02 root image analysis software (Dianjiang Technology Co. Ltd., Shanghai, China, 2022). SRL (cm g^−1^) was calculated as the ratio of root length to dry biomass, while SRA (cm^2^ g^−1^) was derived by dividing total surface area by biomass. RTD (g cm^−3^) was determined as dry weight per unit volume, and RDMC (g g^−1^) was computed as the ratio of dry to fresh mass.

**TABLE 1:** Root traits and their ecological significance.

Trait index	Abbreviation	Unit	Ecological significance
Root length	RL	cm	Reflects the root resource acquisition
Root diameter	RD	mm	Reflects the root resource acquisition and physiological function
Root volume	RV	cm^3^	Reflects the root resource acquisition
Specific root length	SRL	cm g^−1^	Reflect the root uptake area at a given biomass cost, which indicates the nutrient acquisition capacity
Specific root area	SRA	cm^2^ g^−1^	Reflect the root uptake area at a given biomass cost, which indicates the nutrient acquisition capacity
Root tissue density	RTD	g cm^−3^	Reflects the root resource acquisition and defense capability
Root dry matter content	RDMC	g g^−1^	Reflects the ability of root system to acquire soil resource, and the tissue investment
Root carbon concentration	C	mg g^−1^	Affects the root resource acquisition and metabolic rate
Root nitrogen concentration	N	mg g^−1^	
Root phosphorus concentration	P	mg g^−1^	
Root potassium concentration	K	mg g^−1^	
Root calcium concentration	Ca	mg g^−1^	
Root magnesium concentration	Mg	mg g^−1^	
Nitrogen phosphorus ratio	N:P		Nutrient limitation indicator

Dried fine roots were ground into a homogeneous powder for subsequent determination of root chemical traits. The C and N concentrations were analyzed using a Dumas‐type combustion C–N elemental analyzer (Vario MAX CN, Elementar Analysen‐systeme GmbH, Hanau, Germany), while P, K, Ca, and Mg were measured by an inductively coupled plasma atomic‐emission spectrometer (iCAP 7400, Thermo Fisher Scientific, Bremen, Germany). The N:P ratio was calculated as an indicator of nutrient limitation (Wang et al. 2019).

### 2.4 Data Analyses

The mean value of fine root traits was derived from three intact root systems sampled from individual plants. Prior to statistical analysis, all data were log_10_‐transformed. Trait variations were analyzed using independent‐sample *t*‐test (*stats* package), while trait correlations were examined through Pearson's correlation analysis (*Hmisc* package). Principal component analysis (PCA, *vegan package*) was employed to assess multivariate trait associations. All statistical procedures were performed using R software (R Core Team 2024).

## 3 Results

**FIGURE 2:**
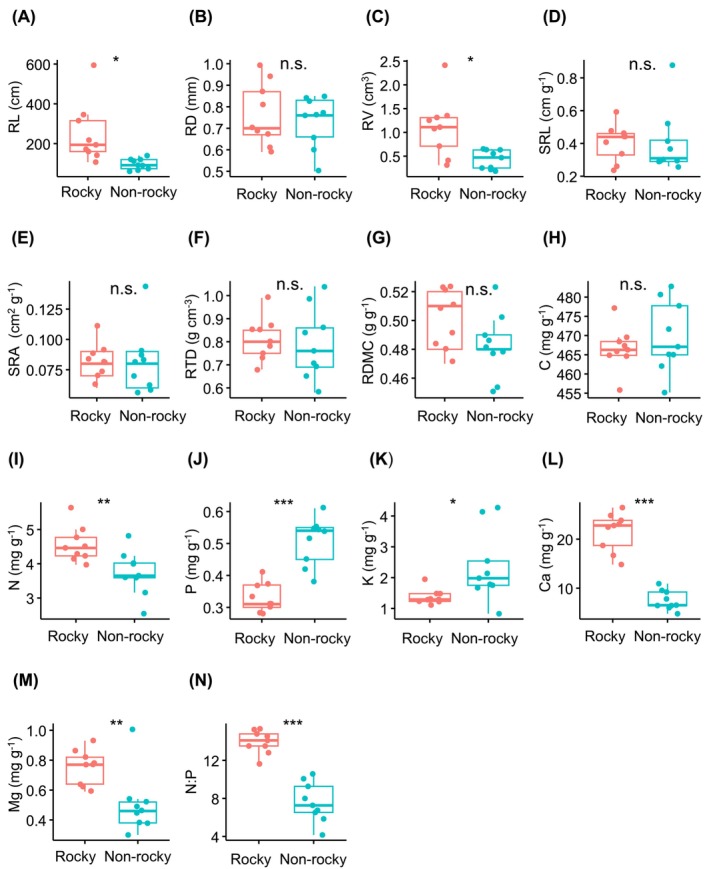
T test comparing fine root traits between forests with rocky and nonrocky desertification. Traits include root length (RL), root diameter (RD), root volume (RV), specific root length (SRL), specific root area (SRA), root tissue density (RTD), root dry matter content (RDMC), carbon (C), nitrogen (N), phosphorus (P), potassium (K), calcium (Ca), magnesium (Mg), and the N:P ratio. *p < 0.05; **p < 0.01; ***p < 0.001.

Fine root morphological analysis showed significantly greater RL and RV in rocky desertification forests compared to nonrocky desertification forests (Figure 2A,C; Table 2). However, no significant differences were observed in RD, SRL, SRA, RTD, or RDMC between the two forest types (Figure 2B,D–G; Table 2). Regarding nutrient concentrations, roots in rocky desertification forests exhibited significantly higher N, Ca, Mg levels, and N:P ratios, whereas P and K concentrations were notably elevated in nonrocky desertification forests (Figure 2I−N; Table 2).

**TABLE 2:** Mean value (±SE) of fine root traits between forests with rocky and nonrocky desertification.

Traits	Forests with rocky desertification	CV (%)	Forests with nonrocky desertification	CV (%)
RL	249.85 ± 47.44	60.42	97.34 ± 8.61	28.16
RD	0.76 ± 0.05	18.97	0.73 ± 0.04	16.40
RV	1.10 ± 0.20	56.34	0.43 ± 0.06	46.49
SRL	0.40 ± 0.04	28.08	0.40 ± 0.06	48.38
SRA	0.08 ± 0.01	17.91	0.08 ± 0.01	31.09
RTD	0.81 ± 0.03	11.29	0.79 ± 0.05	19.53
RDMC	0.50 ± 0.01	4.00	0.48 ± 0.01	4.61
C	466.65 ± 1.75	1.19	469.70 ± 2.89	1.96
N	4.56 ± 0.16	11.30	3.73 ± 0.20	17.40
P	0.33 ± 0.01	13.72	0.51 ± 0.02	14.57
K	1.37 ± 0.08	18.01	2.34 ± 0.36	49.02
Ca	21.51 ± 1.22	18.09	7.53 ± 0.62	26.36
Mg	0.75 ± 0.04	15.25	0.50 ± 0.06	40.66
N:P	13.97 ± 0.39	8.87	7.63 ± 0.66	27.41

In rock desertification forest ecosystems, root N concentration exhibited a significant positive correlation with both P (*r* = 0.70, *p* < 0.05) and K (*r* = 0.70, *p* < 0.05) concentrations (Figure 2A). Additionally, root P concentration was positively associated with SRL (*r* = 0.70, *p* < 0.05) and SRA (*r* = 0.70, *p* < 0.05). In contrast, within nonrocky desertification forests, root C concentration showed a strong negative correlation with Ca (*r* = −0.70, *p* < 0.05) and Mg (*r* = −0.90, *p* < 0.01) concentrations (Figure 2B). Furthermore, SRL was significantly negatively correlated with RV (*r* = −0.70, *p* < 0.05), RD (*r* = −0.99, *p* < 0.01), and RTD (*r* = −0.70, *p* < 0.05). Similarly, SRA displayed strong negative relationships with RV (*r* = −0.70, *p* < 0.05), RD (*r* = −0.90, *p* < 0.05), and RTD (*r* = −0.90, *p* < 0.05).

Principal component analysis (PCA) performed on 14 fine root traits showed that the first two principal components accounted for 60.27% and 16.92% of the total variance, respectively (Figure 4). The first principal axis exhibited negative correlations with RL and RV, whereas the second axis demonstrated positive correlations with SRA, SRL, root Ca concentration, and N:P ratio, but negative correlations with root P and K concentrations. Notably, forests affected by rocky desertification displayed positive relationships with higher values of RV, RL, SRL, SRA, root Ca, and N concentrations, suggesting an adaptive resource‐acquiring strategy. Conversely, forests in nonrocky desertification areas were positively correlated with elevated root P and K concentrations, indicative of a resource‐conserving strategy. The clear separation between rocky and nonrocky desertification samples in the multivariate space underscores their distinct resource utilization strategies (Figure 4).

## 4 Discussion

Fine roots are essential for water and nutrient uptake from the soil, serving as a critical interface between plants and their surrounding environment (Guo et al. 2008; Ma et al. 2018; Sun et al. 2021). Their functional traits, encompassing both morphological and chemical properties, reflect adaptive strategies to varying environmental conditions (Bardgett et al. 2014; Hodge et al. 2009). Key morphological traits—including root length, root volume, root diameter, specific root length, specific root area, root tissue density, and root dry matter content—serve as vital indicators of these adaptations (Addo‐Danso et al. 2020; Ma et al. 2018; Sun et al. 2021). In this study, forests affected by rocky desertification exhibited significantly greater root length and root volume compared to those in nondesertified areas (Figure 1). This suggests that plants in rocky desertification environments develop more extensive root systems to mitigate water scarcity and nutrient‐deficient soils (Lõhmus et al. 2006; Ostonen et al. 2011; Pinno and Wilson 2013).

Root diameter, a trait linked to stress resistance, plays a crucial role in resource acquisition and environmental adaptation (Chapin III 1974; Guo et al. 2008; Lugli et al. 2020). Fine roots, essential for water and nutrient absorption, also contribute to plants' resilience to harsh conditions and resilience (Cusack et al. 2021; Kou et al. 2015; Lugli et al. 2020). Specific root length and specific root area are key indicators of nutrient uptake efficiency, with higher specific root length values associated with enhanced absorptive capacity (Metcalfe et al. 2008). Conversely, root tissue density and root dry matter content reflect conservative growth strategies, whereas lower values suggest a resource‐acquisitive strategies approach favoring rapid growth (Ostonen et al. 2013; Freschet et al. 2021). Interestingly, no significant differences were detected in root diameter, specific root length, specific root area, root tissue density, and root dry matter content between rocky and nonrocky desertification forests. This uniformity implies that these traits may be phylogenetically conserved rather than shaped by environmental pressures (Aritsara et al. 2022; Comas et al. 2012; Gao et al. 2024; Sun et al. 2021).

Chemical traits, particularly root nutrient concentrations (C, N, P, K, Ca, and Mg), play a crucial role in plant growth and productivity (Bowsher et al. 2016; Guo et al. 2008; Majdi and Viebke 2004; Pan et al. 2022). In our study, rocky desertification forests exhibited higher root N, Mg, and Ca concentrations but lower P concentrations compared to nonrocky desertification forests. The elevated light intensity in rocky desertification areas likely enhances photosynthesis activity, increasing Mg demand for chlorophyll synthesis while promoting N and Ca accumulation due to altered soil mineral dynamics (Bai et al. 2024; Hu et al. 2020; Ji et al. 2009; Li et al. 2019). Notably, root P concentration was significantly reduced in rocky desertification forests, which we attribute to lower soil total P, available P, and occluded P levels, coupled with higher iron‐bound and calcium‐bound P fractions. These soil P dynamics limit P acquisition by vegetation, subsequently affecting P concentration in plant tissues (leaves, roots, and litter) (Liu et al. 2023). Furthermore, fine roots in rocky desertification forests displayed a significantly higher N:P ratio than those in nonrocky desertification forests. This pattern may reflect nutrient‐limiting conditions in rock desertification ecosystems, where fine roots prioritize P allocation to rRNA for protein synthesis, thereby optimizing nutrient and water uptake efficiency (Lambers, Iii, and Pons 2008; Matzek and Vitousek 2009; Tang et al. 2018). Paradoxically, this adaptation leads to a lower N:P ratio in fine roots, highlighting a key physiological response to resource scarcity in these environments.

The N:P ratio in fine roots is a critical indicator of nutrient limitation, where values below 14 suggest N limitation, values above 16 indicate P limitation, and ratios between 14 and 16 reflect a balanced N and P availability (Wang et al. 2019). Our findings demonstrate N limitation in both rocky desertification (13.97) and nonrocky desertification (7.63) forests, which contrast with our previous leaf‐based assessment showing P limitation in these forest types (leaf N:P ratios: 18.47 and 16.04 for rocky and nonrocky desertification, respectively; Bai et al. 2024). This inconsistency likely reflects differential nutrient requirements between leaves and roots under varying environmental conditions, highlighting the importance of considering both foliar and root nutrient dynamics in rocky desertification vegetation restoration strategies.

**FIGURE 3:**
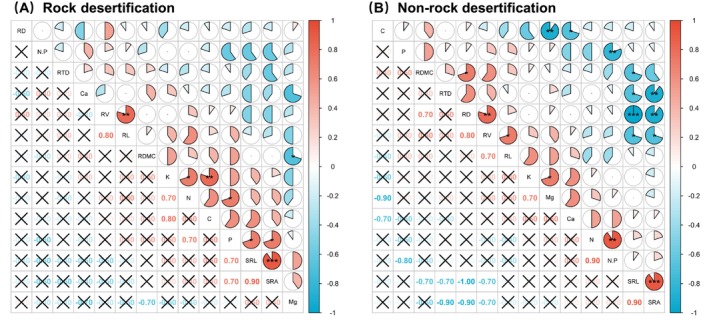
Pearson's correlation between fine root traits of *Quercus rehderiana* in forests with rocky (A) and nonrocky (B) desertification. Traits include root length (RL), root diameter (RD), root volume (RV), specific root length (SRL), specific root area (SRA), root tissue density (RTD), root dry matter content (RDMC), carbon (C), nitrogen (N), phosphorus (P), potassium (K), calcium (Ca), magnesium (Mg), and the N:P ratio. *p < 0.05; **p < 0.01; ***p < 0.001.

Fine root functional traits reflect a trade‐off between resource acquisition and conservation strategies (Grassein et al. 2015; Weemstra et al. 2020). In rocky desertification forests, our findings revealed a significant positive correlation between SRL, SRA, and root P concentration. This relationship likely arises because, under P‐deficient soil conditions typical of these ecosystems (Liu et al. 2023), plants enhance SRA and SRL to optimize P acquisition. Increased P uptake facilitates ATP synthesis, rRNA production, and protein synthesis, thereby improving nutrient and water absorption efficiency (Lambers, Iii, and Pons 2008; Lambers, Raven, et al. 2008; Wright et al. 2005). Furthermore, P absorption enhances K^+^ uptake, which plays a vital role in stress tolerance (e.g., drought, high temperature, salinity) by preserving chloroplast integrity, optimizing light absorption, and enhancing photosynthetic CO_2_ assimilation (Egilla et al. 2005; Tränker et al. 2018). K^+^ also regulates stomatal opening, maintaining transpiration rates and ensuring efficient gas exchange, water transport, and nutrient utilization (Sardans and Peñuelas 2021). These adaptive mechanisms highlight how fine roots in rock desertification forests adjust to arid, nutrient‐poor environments through synergistic traits–elemental interactions. In contrast, nonrock desertification forests exhibited significant negative correlations between specific root area/specific root length and root diameter, root tissue density, and root volume. In arid, infertile soils, plants reduce root diameter, root tissue density, and root volume to enhance stress resistance, whereas in moist, fertile soils, they prioritize specific root length and specific root area to maximize resource acquisition (Craine et al. 2001; Kramer‐Walter et al. 2016; Lõhmus et al. 1989; Ostonen et al. 2007). Additionally, root C concentration showed negative correlations with Ca and Mg concentrations, suggesting that elevated Ca or Mg levels may constrain C storage and reduce primary productivity (Zhao et al. 2000). These findings underscore how fine roots in nonrock desertification forests employ trait trade‐offs to adapt to varying environmental conditions. Principal component analysis (PCA) further indicated that *Quercus rehderiana* in rocky desertification forests adopted a resource‐conserving strategy to cope with arid and nutrient‐limited soils (Figure 3), aligning with prior studies in karst regions (Pan et al. 2018; Zhou 2015). In these regions, slow growth rates and high root efficiency are characteristics adapted to chronic nutrient stress (Elser et al. 2007; Lambers, Raven, et al. 2008). This “conservation strategy” likely reflects long‐term evolutionary pressures in these ecosystems, where nitrogen limitation intensifies competition for phosphorus, thereby driving organisms to evolve traits that optimize phosphorus utilization beyond rapid growth (Vitousek and Howarth 1991; Reich 2014). Notably, alternative nutrient acquisition strategies mediated by soil microbial activity−independent of root symbiotic components—have been documented. Rhizosphere microbial activity correlates positively with the “fast‐slow” plant resource gradient, where fast‐growing plants exhibit higher microbial metabolic activity than slow‐growing ones (Han et al. 2023). Thus, future research integrating leaf, stem, root, and soil microbial analyses will provide a more comprehensive understanding of plant adaptive strategies across diverse environments.

**FIGURE 4:**
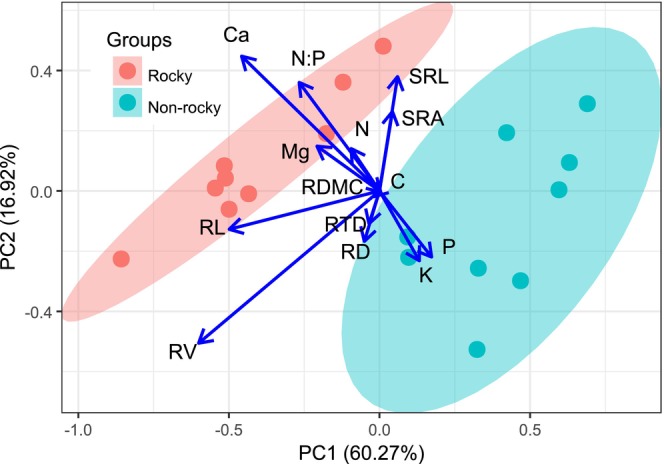
Biplot of the first two principal component axes (PCA) illustrating the relationships between fine root traits and the loadings of nine individuals from forests with rocky desertification and nine individuals from forests with nonrocky desertification. Traits include root length (RL), root diameter (RD), root volume (RV), specific root length (SRL), specific root area (SRA), root tissue density (RTD), root dry matter content (RDMC), carbon (C), nitrogen (N), phosphorus (P), potassium (K), calcium (Ca), magnesium (Mg), and the N:P ratio. All leaf traits were log_10_‐transformed prior to analysis.

## 5 Conclusions

We examined 14 fine root traits of *Quercus rehderiana* in both rocky and nonrocky desertification forests. Significant variations were detected in both morphological traits and nutrient concentrations between the two forest types. Specifically, rocky desertification forests exhibited higher root length, root volume, and concentrations of root N, Ca, and Mg, whereas nonrocky desertification forests displayed elevated P and K levels. Furthermore, we identified both synergistic and trade‐off relationships among these traits. In rock desertification forests, SRL and SRA were positively correlated with root P, while root N showed positive associations with root P and K, suggesting a synergistic nutrient acquisition strategy. Conversely, in nonrocky desertification forests, specific root length and specific root area were negatively correlated with root tissue density, root diameter, and root volume, and root C was inversely related to Ca and Mg concentrations, reflecting a trade‐off in resource allocation. These findings indicate that *Quercus rehderiana* fine roots adopt a resource‐acquiring strategy in rocky desertification environments to cope with nutrient and water limitations. Overall, the species demonstrates flexible resource allocation strategies across different habitats, highlighting its strong adaptability—a key characteristic for ecological restoration in rocky desertification regions.

## Author Contributions

**Xiaolong Bai:** investigation (equal), methodology (equal), writing – original draft (equal), writing – review and editing (equal). **Shun Zou:** investigation (equal), methodology (equal). **Wanchang Zhang:** writing – review and editing (equal). **Tu Feng:** investigation (equal), methodology (equal). **Dongpeng Lv:** methodology (equal). **Bin He:** investigation (equal). **Wangjun Li:** conceptualization (equal), methodology (equal), writing – review and editing (equal).

## Conflicts of Interest

The authors declare no conflicts of interest.

## Data Availability

Data files and README file will be available via Dryad: https://doi.org/10.5061/dryad.vhhmgqp4r.
